# Shikonin-induced necroptosis is enhanced by the inhibition of autophagy in non-small cell lung cancer cells

**DOI:** 10.1186/s12967-017-1223-7

**Published:** 2017-05-31

**Authors:** Hyo-Jin Kim, Ki-Eun Hwang, Do-Sim Park, Seon-Hee Oh, Hong Young Jun, Kwon-Ha Yoon, Eun-Taik Jeong, Hak-Ryul Kim, Young-Suk Kim

**Affiliations:** 1 Department of Internal Medicine, Institute of Wonkwang Medical Science, Wonkwang University, School of Medicine, Iksan, Jeonbuk South Korea; 2 Department of Laboratory Medicine, Wonkwang University, School of Medicine, Iksan, Jeonbuk South Korea; 3 Department of Premedicine, Chosun University, School of Medicine, Gwangju, South Korea; 4 Imaging Science Research Center, Wonkwang University Hospital, Iksan, Jeonbuk South Korea; 5 Department of Radiology, Wonkwang University, School of Medicine, Iksan, Jeonbuk South Korea

**Keywords:** Shikonin, Necroptosis, Autophagy, Non-small cell lung cancer, RIP1

## Abstract

**Background:**

Shikonin, a natural naphthoquinone pigment purified from *Lithospermum erythrorhizon*, induces necroptosis in various cancer types, but the mechanisms underlying the anticancer activity of shikonin in lung cancer are not fully understood. This study was designed to clarify whether shikonin causes necroptosis in non-small cell lung cancer (NSCLC) cells and to investigate the mechanism of action.

**Methods:**

Multiplex and caspase 8 assays were used to analyze effect of shikonin on A549 cells. Cytometry with annexin V/PI staining and MTT assays were used to analyze the mode of cell death. Western blotting was used to determine the effect of shikonin-induced necroptosis and autophagy. Xenograft and orthotopic models with A549 cells were used to evaluate the anti-tumor effect of shikonin in vivo.

**Results:**

Most of the cell death induced by shikonin could be rescued by the specific necroptosis inhibitor necrostatin-1, but not by the general caspase inhibitor Z-VAD-FMK. Tumor growth was significantly lower in animals treated with shikonin than in the control group. Shikonin also increased RIP1 protein expression in tumor tissues. Autophagy inhibitors, including methyladenine (3-MA), *ATG5* siRNA, and bafilomycin A, enhanced shikonin-induced necroptosis, whereas *RIP1* siRNA had no effect on the apoptotic potential of shikonin.

**Conclusions:**

Our data indicated that shikonin treatment induced necroptosis and autophagy in NSCLC cells. In addition, the inhibition of shikonin-induced autophagy enhanced necroptosis, suggesting that shikonin could be a novel therapeutic strategy against NSCLC.

## Background

Under normal conditions, programmed cell death (PCD) maintains a balance between cell death with survival; however, once the equilibrium becomes disturbed, PCD plays a key role in determining the fate of cancer cells [1, 2]. “Necroptosis” is a type of PCD, which is characterized by the swelling of the cytosol and organelles and rupture of the plasma membrane, with a subsequent loss of the intracellular contents.

Autophagy involves in cellular homeostasis and differentiation, as well as in tissue remodeling, aging, cancer, and other diseases. This process can be activated by a lack of nutrients and growth factors in the extracellular microenvironment, but it can also contribute to programmed cell death under specific environmental conditions [3–5]. Autophagy is an important, well-established cell survival mechanism, especially in cells under stress conditions, such as starvation [6, 7]. On the other hand, autophagy has been implicated in the cell death process, either in apoptosis, or in non-apoptotic or necrotic cell death, including autophagic cell death [8].

Necrosis is another programmed and regulated cell death process [9]. Among various forms of necrotic cell death, necroptosis and PARP-mediated necrosis have emerged as two important forms, and our understanding of the molecular mechanisms and biological functions of these processes has increased. Necroptosis refers to a specific form of caspase-independent, non-apoptotic or necrotic cell death that is triggered by cell death ligands (TNF-α and FasL) via cell death receptors and a unique downstream signaling pathway [10, 11]. At present, the relationship between autophagy and necroptosis is rather complex.

Shikonin, purified from *Lithospermum erythrorhizon*, has been used for thousands of years in traditional Chinese medicine for the treatment of burns, carbuncles, measles, macular eruptions, and sore throat [12, 13]. Recent studies have demonstrated that shikonin has significant anti-tumor potential, inducing apoptosis and necroptosis in cancer cell lines of various types, including breast cancer, hepatocellular carcinoma, glioma, osteosarcoma, and leukemia [14–17]. However, the effect on lung cancer cell lines remains unknown. In this study, we hypothesized that autophagy plays an important role in the outcome of necroptosis following shikonin treatment. Furthermore, we tested whether shikonin can induce necroptosis and autophagy in non-small cell lung cancer (NSCLC) cells and whether the inhibition of autophagy can drive lung cancer cells to necroptosis.

## Methods

### Materials

Roswell Park Memorial Institute Medium 1640 (RPMI 1640), fetal bovine serum, and antibiotics (penicillin and streptomycin) were obtained from GIBCO BRL Co. (Grand Island, NY, USA). Shikonin, 3-(4,5-dimethyl-2-thiazolyl)-2,5-diphenyl-2*H*-tetrazolium bromide (MTT), propidium iodide (PI), and dimethyl sulfoxide were purchased from Sigma-Aldrich (St. Louis, MO, USA). Primary antibodies against caspase-3, poly(ADP-ribose) polymerase (PARP), AMPK, mTOR, Beclin, ATG5, LC3B, p62/SQSTM1, and GFP were purchased from Cell Signaling Technology (Beverly, MA, USA). Antibodies against RIP1 were purchased from Abcam, Inc. (Cambridge, UK). 3-Methyl adenine (3-MA) and bafilomycin A were purchased from Sigma-Aldrich. Caspase inhibitor Z-VAD-FMK, Z-DEVD-FMK, Z-IETD-FMK, and Z-LEHD-FMK were purchased from R&D Systems (Minneapolis, MN, USA). Necrostatin-1 (Nec-1) was purchased from Tocris Bioscience (Ellisville, MO, USA). Anti-rabbit IgG-conjugated horseradish peroxidase (HRP) antibodies and enhanced chemiluminescence (ECL) kits were purchased from Amersham Pharmacia Biotech (Buckinghamshire, UK).

### Cell culture

A549 human lung cancer cells were obtained from the Korean Cell Line Bank (Seoul, Korea) and grown in RPMI 1640 containing 100 units/mL penicillin, 0.1 mg/mL streptomycin, and 10% fetal bovine serum. The cells were incubated in a humidified atmosphere of 5% CO_2_ in air at 37 °C and maintained in log-phase growth.

### Multiplexed viability and cytotoxicity assays

The CellTox Green Cytotoxicity Assay (Promega, Madison, WI, USA) was used to measure the DNA in dead cells and the CellTiter-Glo 2.0 Assay (Promega) was then used to measure ATP as a marker of viable cells. A549 cells were seeded in 96-well plates at 20,000 cells/well in 50 µL and incubated at 37 °C for 24 h in a 5% CO_2_ incubator, followed by the addition of 50 μL/well CellTox Green Cytotoxicity Assay Reagent (Promega). After 15 min of incubation at room temperature, the resulting fluorescence was measured in the 485Ex 530Em channels using a SpectraMax M3 Plate Reader. After the readings were obtained, 100 µL of CellTiter-Glo 2.0 assay reagent was added to all wells and the resulting luminescence was measured using a SpectraMax M3 Plate Reader after 10 min of incubation at 37 °C.

### Caspase 8 assay

A549 cells were dispensed in culture medium at 2 × 10^4^ cells/well in white-walled 96-well luminometer plates and incubated for 24 h at 37 °C in the presence or absence of test materials, followed by the addition of 100 μL/well Caspase-Glo 8 Reagent (Promega). After 30 min of incubation at room temperature, the luminescence intensity was measured using a SpectraMax M3 Plate Reader.

### MTT assay

After cells were treated with the specified drugs, MTT was added to the cell suspension and incubated for 4 h. The cells were then washed three times with phosphate-buffered saline (PBS; pH 7.4), and the insoluble formazan product was dissolved in dimethyl sulfoxide. The optical density (OD) at 595 nm in each well was measured using a microplate reader (Titertek Multiskan; Flow Laboratories, North Ryde, New South Wales, Australia). The OD resulting from formazan production in control cells was defined as 100% cell viability, and all other measurements were expressed as a percentage of the control cell value.

### Annexin V/PI assay

Annexin V-FITC and PI staining were used to analyze whether A549 cells were undergoing early/late apoptosis or necroptosis. Cells in the exponential growth phase (2.5 × 10^5^ cells) were seeded in 35-mm^2^ dishes and were incubated at 37 °C for the indicated times in the presence or absence of specified test drugs. The cells, both adherent and floating, were then harvested and analyzed by the annexin V assay according to the manufacturer’s instructions. Pelleted cells were briefly washed with PBS and resuspended in annexin-binding buffer. They were then incubated with annexin V-FITC and PI for 15 min at room temperature. After incubation, the stained cells were analyzed using a FACSCalibur system and Cell Quest software (Becton–Dickinson, San Jose, CA, USA).

### Tumor xenograft studies in nude mice

Five- to six-week-old BALB/c athymic nude mice (Charles River, Tokyo, Japan) were housed in cages with HEPA-filtered air (12-h light/dark cycle). Food and autoclaved water were provided ad libitum. A549 cells were injected subcutaneously (s.c.) into both hind legs of each mouse. When the implanted tumors reached a volume of 90–130 mm^3^, the mice were randomly assigned to one of two groups (n = 5 per group). For the experimental group, shikonin (2.0 mg/kg, diluted PBS) was injected intraperitoneally once per da. The control group received PBS alone. Animals were monitored for 14 days or until the tumors reached a volume of 1300 mm^3^.

### Tumor orthotopic studies in nude mice

Nude mice were intraperitoneally injected with thiopental sodium (0.08 mL/kg of body weight) to induce anesthesia; subsequently, the mice were placed in the right lateral decubitus position. Then, 50 μL of the A549 single cell suspension (1.5 × 10^6^) prepared using a 1-mL injector was rapidly inoculated percutaneously into the upper margin of the sixth intercostal rib on the left anterior axillary line to a depth of about 5 mm. The needle was then promptly removed. The mice were maintained in the left lateral decubitus position after injection and were observed until complete recovery.

### Micro-CT imaging analysis

Mice with lung cancer underwent micro-CT scanning weekly after cell inoculation. Whole lungs were scanned for the detection of tumors at 20× magnification. For each tumor, micro-CT images were used to reconstruct three-dimensional images (axial, coronal, and 3D). Tumor size was evaluated using imaging software (Xelis; Infinitt, Seoul, Korea). The diameter of the tumor was defined as the maximum diameter of the tumor in a 2D plane. The tumor volume was evaluated using volume analysis software (VGStudio MAX, Heidelberg, Germany). Small tumors were not included in the data analysis owing to the inability to measure the size of these tumors.

### H&E staining

At the end of the study, the whole tumor was harvested from each mouse and fixed in 100 mL of buffered formalin for 24 h. Formalin-fixed tissue was paraffin-embedded, sectioned (3–5 μm), and stained with H&E. Sections were evaluated for necrotic degree of tumors and counted under a microscope.

### Acridine orange staining

Autophagy is characterized by the formation and promotion of acidic vesicular organelles (AVOs). In acridine orange-stained cells, the cytoplasm and nucleus exhibit bright green and dim red fluorescence, whereas acidic compartments exhibit bright red or orange fluorescence, as described previously [18]. Following drug treatment, 5 µg/mL acridine orange (A1301; Invitrogen, Carlsbad, CA, USA) was added to the culture medium, and the cells were incubated at 37 °C for 15–30 min. The cells were then trypsinized, washed twice with cold PBS, and observed under a confocal microscope. Fluorescence imaging was performed using a blue bandpass filter with 490 nm excitation, and the fluorescence of the green and orange channels was recorded and merged.

### Transient transfection

Adenoviral GFP-LC3B was kindly provided by Dr. Xiao-Ming Yin (University of Pittsburgh School of Medicine, Pittsburgh, PA, USA). After the cells were washed with OPTI-MEM (Invitrogen), DNA was transfected into cells using Lipofectamine™ 2000 according to the manufacturer’s protocol (Invitrogen). After incubation for 4 h, the medium was replaced with complete medium containing 10% serum and antibiotics. The cells were incubated for 24 h and treated as indicated in the figure legends. Images were obtained using a confocal microscope (FluoView™ FV1000; Olympus, Tokyo, Japan).

### Western blotting

Cells were harvested and lysed in radioimmunoprecipitation assay buffer [50 mM Tris–Cl (pH 7.4), 1% NP40, 150 mM NaCl, 1 mM EDTA, 1 mM phenylmethylsulfonyl fluoride, 1 µg/mL each of aprotinin and leupeptin, and 1 mM Na_3_VO_4_]. After centrifugation at 12,000×*g* for 30 min, the supernatant was collected, and the protein concentration was determined by the Bradford method (Bio-Rad Protein Assay; Hercules, CA, USA). Equal amounts of protein were separated by 10% sodium dodecyl sulfate-polyacrylamide gel electrophoresis (SDS-PAGE) under reducing conditions and were subsequently transferred to polyvinylidene difluoride membranes. The membranes were blocked with 5% skim milk in TBS-T [25 mM Tris (pH 7.6), 138 mM NaCl, and 0.05% Tween-20] for 1 h and probed with primary antibodies (at 1:1000–1:5000). After washing, the membranes were incubated with the relevant HRP-conjugated secondary antibody (at 1:2000–1:10,000). Immunoreactive signals were detected using an ECL detection system.

### Gene silencing

Pooled small interfering RNA (siRNA) oligonucleotides against *ATG5* were purchased from Cell Signaling Technology. siRNA against *RIP1* was purchased from Ambion (Austin, TX, USA). Cells were transfected with 100 nM pooled oligonucleotide mixture using Lipofectamine2000 (Invitrogen) according to the manufacturer’s protocol. Twenty-four hours after transfection, media were removed and cells were treated with the indicated drugs. Gene silencing efficacy by siRNA was assessed by a western blot analysis.

### Statistical analysis

Each experiment was performed at least three times, and all values are expressed as the mean ± SD of triplicate samples. The Student’s *t* test was used to determine statistical significance. Values of *p* < 0.05 were considered statistically significant.

## Results

### Effect of shikonin on caspase 8 is independent of cytotoxicity in A549 cells

**Fig. 1:**
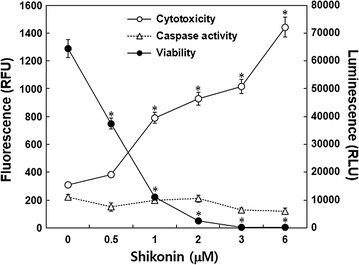
Effect of shikonin on the viability, cytotoxicity, and caspase 8 activity of A549 cells. Cells were treated with various concentrations of shikonin for 24 h. Viability and cytotoxicity were then measured by a multiplex assay, and caspase 8 activity was evaluated by a caspase 8 assay. Values are presented as mean ± SD of three independent experiments. *p < 0.05 compared to control

The effect of shikonin on A549 cells was determined by a multiplex assay after treatment with various concentrations of shikonin for 24 h. Shikonin increased cytotoxicity and decreased viability in a dose-dependent manner. In contrast, shikonin treatment had no effect on the activation of caspase 8 (Fig. 1).

### Effect of shikonin on necroptosis in A549 cells

**Fig. 2:**
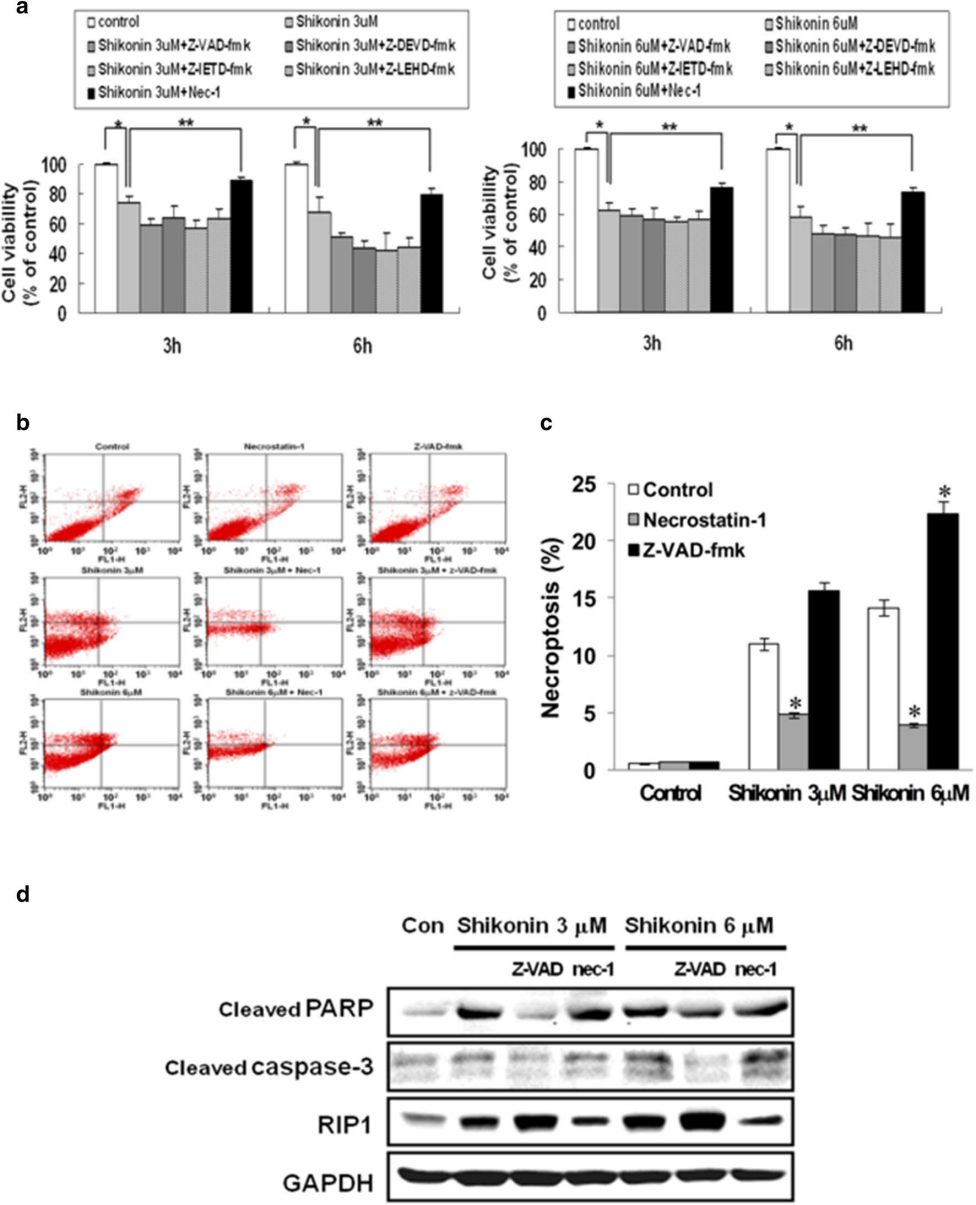
Cell death induced by shikonin in A549 cells occurs by necroptosis. **a** Cells were pretreated for 2 h in the presence or absence of Nec-1 (100 μM), Z-VAD (20 μM), Z-DEVD (20 µM), Z-IETD (10 µM), or Z-LEHD (20 µM) and then incubated with shikonin (3 or 6 µM) for 3 or 6 h. Viability was then measured by the MTT assay. Values are represented as the mean ± SD of three independent experiments. *p < 0.01 compared with the control, **p < 0.05 compared with the shikonin group. **b** Cells were pretreated for 2 h in the presence or absence of Nec-1 (100 µM) or Z-VAD (20 µM) and incubated with shikonin for 3 h. Necroptosis was evaluated by annexin V/PI staining. **c** Values are presented as mean ± SD of three independent experiments. *p < 0.05 compared to control. **d** Cells were pretreated for 2 h in the presence or absence of Nec-1 (100 µM) or Z-VAD (20 µM) and then treated with shikonin (3 or 6 μM) for 3 h. The cells were then lysed, and the cell lysate was subjected to 10 and 15% SDS-PAGE to measure the expression of the indicated proteins. The levels of RIP1, cleaved PARP, and cleaved caspase 3 were measured by western blotting. Data are representative of two independent experiments

To investigate the mechanism by which shikonin killed A549 cells, inhibitors of apoptosis and necroptosis were added prior to shikonin treatment. As shown in Fig. 2a, pretreatment with Nec-1 protected the viability of A549 cells treated with 3 and 6 µM shikonin. In contrast, pretreatment with Z-VAD-fmk, Z-DEVD-fmk, Z-IETD-fmk, and Z-LEHD-fmk did not affect the viability of A549 cells treated with shikonin at either concentration. These results suggested that shikonin induced cell death in A549 cells via the necroptosis pathway.

To confirm whether the observed cell death was due to enhanced necroptosis, the proportion of necrotic cells was determined by annexin V/PI staining. After treatment with 3 and 6 µM shikonin, the necrosis (Annexin V−/PI+) rates in A549 cells were 11.0 and 14.2%, respectively. However, pretreatment with Nec-1 reduced the percentage of necrotic cells in response to 3.0 and 6.0 µM shikonin to 4.8 and 3.9%, respectively. Interestingly, the percentage of necrotic cells after shikonin treatment increased by pretreatment with Z-VAD-fmk (Fig. 2b, c). To further elucidate the mechanism underlying shikonin-induced necroptosis, cell lysates were evaluated by immunoblotting. Our results showed that shikonin treatment enhanced the expression of RIP1; this increase in expression was greater for cells pretreated with Z-VAD-fmk than in those treated with shikonin alone. Pretreatment with Nec-1 decreased RIP1 expression, but did not affect the levels of cleaved PARP and cleaved caspase-3 (Fig. 2d). These results indicated that the cytotoxicity of shikonin in A549 cells is mediated by necroptosis and not by apoptosis.

### Shikonin suppresses lung cancer tumor growth in vivo

**Fig. 3:**
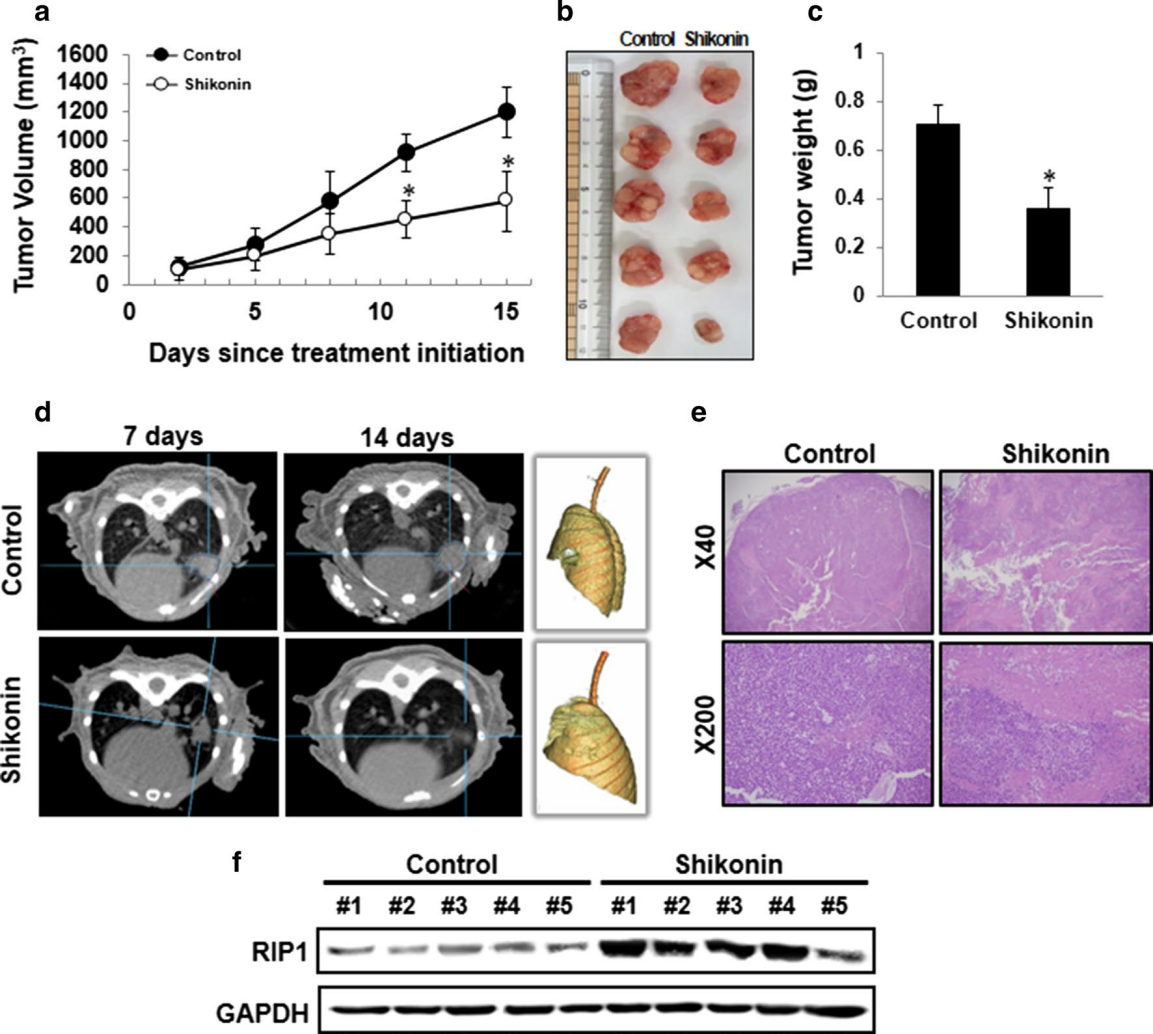
The anti-tumor effect of shikonin on A549 cells by inducing necroptosis in vivo. **a** Athymic nude mice were injected (s.c.) with 2 × 10^6^ A549 cells (0.2 mL cell suspension) in both hind legs. When the implanted tumors reached a volume of 90–130 mm^3^, the mice were randomly divided into two groups with five mice per group. The figure shows the macroscopic appearance of tumors in mice after treatment with shikonin (20 mg/kg, by i.p., once per day) or PBS. Tumor volume was estimated using the following formula: volume = L × W^2^/2. Points, means of five animals; *bars*, SD. *p < 0.01 compared with the control. **b** Image shows the size of tumors at the end of the experiments. **c** The tumors were weighed. The data represent mean ± standard deviation (SD) of five animals. *p < 0.05 compared to the control. **d** CT and 3D images of the orthotopic injection of lung cancer in athymic nude mice. **e** The specimens were fixed in 4% paraformaldehyde for HE staining, as shown at the microscopic level. Images were obtained at ×40 and ×200. **f** Western blot analysis showed that the RIP1 expression levels in tumor tissue was higher in the shikonin group than in the control group. Data are representative of two independent experiments

To demonstrate the anti-tumor effects of shikonin on tumor growth in vivo, athymic nude mice were injected (s.c.) with A549 cells. Animals in the experimental group were injected with shikonin (2.0 mg/kg) intraperitoneally every other day, while those in the control group received PBS. Shikonin treatment significantly reduced tumor growth compared to growth in the control group (Fig. 3a). Furthermore, mice injected with shikonin had significantly smaller tumor volumes and mass compared to those of the control group (Fig. 3b, c). This was confirmed in an orthotopic mouse model, which also showed a decrease in tumor size (Fig. 3d). Based on H&E staining of tumor tissues, the degree of tumor necrosis in the shikonin-treated group was higher than that in the control group (Fig. 3e). In addition, the levels of RIP1 protein in the tumor tissues were higher in the shikonin treatment group than in controls (Fig. 3f).

### Shikonin induced autophagy in A549 cells

**Fig. 4:**
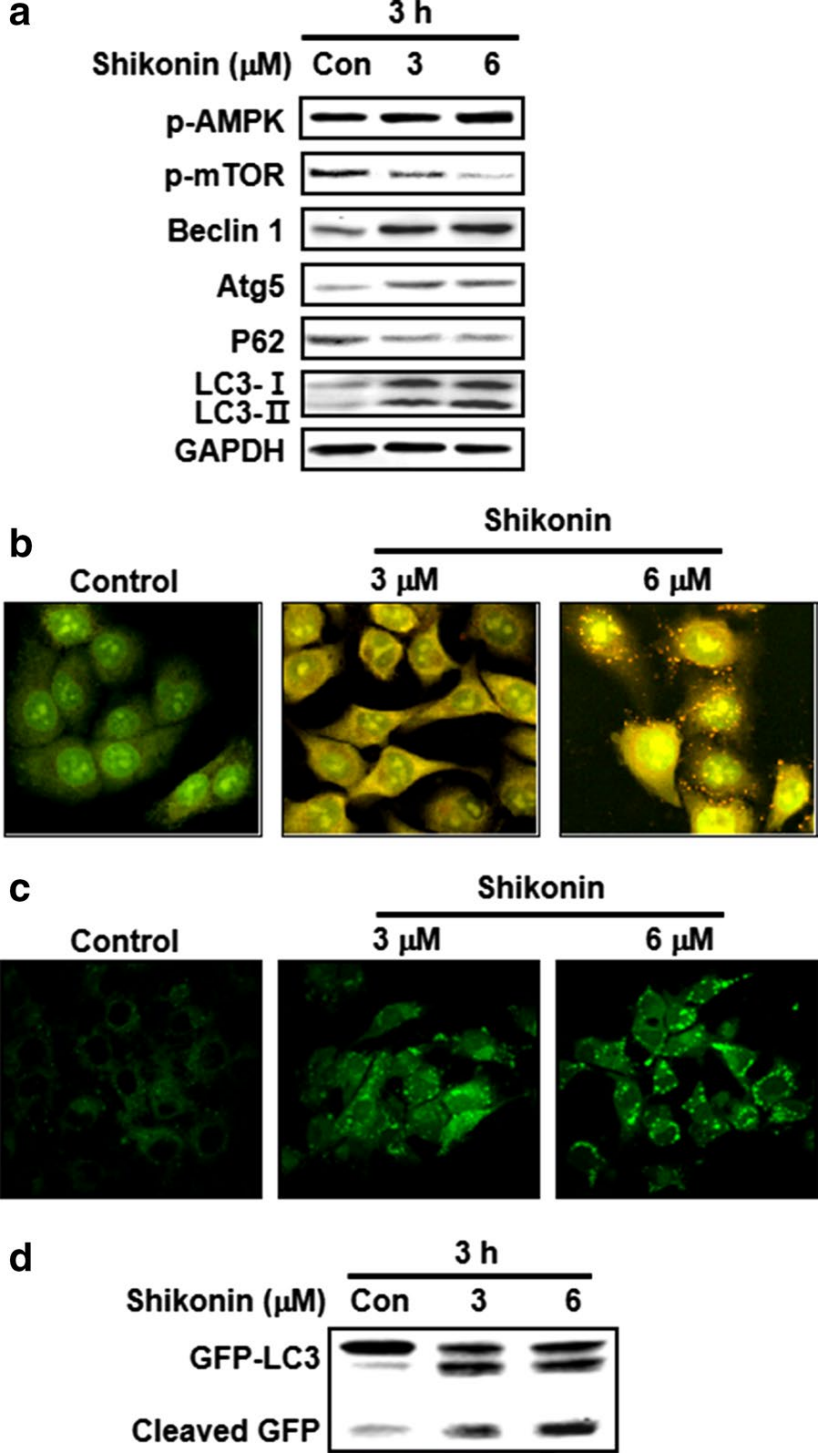
Shikonin-induced autophagy in A549 cells. **a** Cells were treated with 3 or 6 µM shikonin for 3 h, and the levels of the indicated proteins were analyzed by western blotting. Representative images from more than three independent experiments are shown. **b** Acridine orange staining showed lysosomal (*orange*) staining in all cells. Acidic lysosomes in shikonin-treated cells, as observed by confocal microscopy, indicate potential lysosomal activation. **c** Cells were transfected with the GFP-LC3 plasmid for 6 h and then incubated with 3 or 6 µM shikonin for 3 h before analysis by confocal microscopy. Representative images of GFP-LC3-stained shikonin-treated cells are shown (×60). A punctuate pattern of GFP-LC3B indicates autophagosome formation, as observed by confocal microscopy. **d** Western blot analysis was performed using antibodies against GFP. Immunoblots are representative of at least two independent experiments

The effects of shikonin on autophagic cell death in A549 cells were investigated by an immunoblot analysis. Shikonin increased the levels of AMPK phosphorylation, Beclin 1, Atg5, and LC3 in a dose-dependent manner. In contrast, there was a dose-dependent decrease in the phosphorylation of mTOR, a regulator of autophagy, and p62, which is a marker for autophagy (Fig. 4a). Autophagy was also detected by staining lysosomes with acridine orange dye and observing under an inverted microscope to measure fluorescence. Shikonin treatment increased the acidity of the lysosomal compartments compared to that of controls (Fig. 4b).

The induction of autophagy by shikonin was further confirmed by transient transfection of green fluorescence protein (GFP)-LC3B plasmid DNA. In non-treated control cells, a diffuse pattern of GFP fluorescence was observed in the cytoplasm. Shikonin treatment, however, increased the number of LC3-positive GFP puncta compared to that of control cells, and western blotting showed a dose-dependent increase in the amount of free GFP fragments formed by GFP-LC3 degradation (Fig. 4c, d). In conclusion, these data indicate that shikonin induces autophagy in A549 cells.

### *RIP1* siRNA does not affect apoptotic potential induced by shikonin in A549 cells

**Fig. 5:**
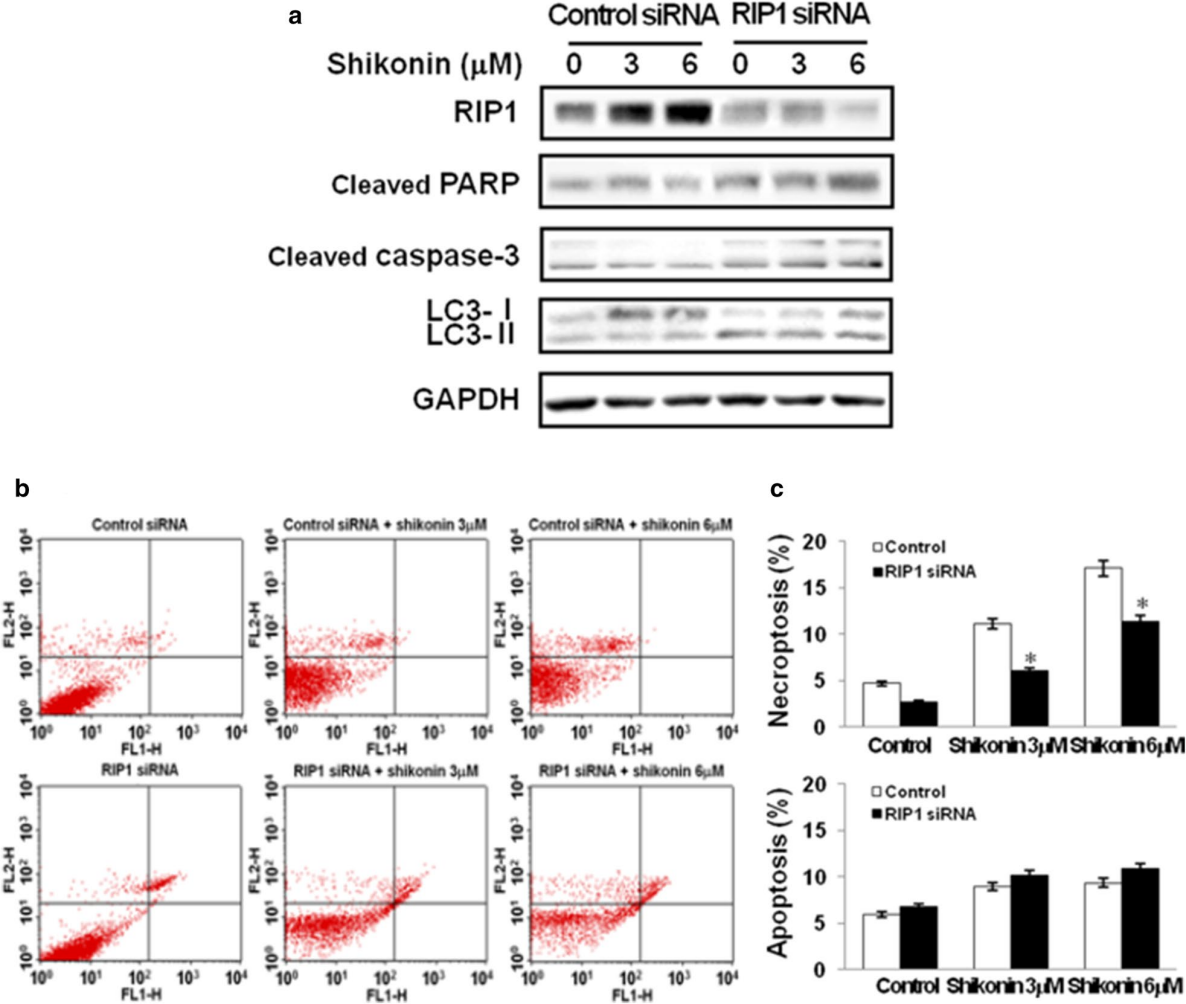
Inhibition of necroptosis does not affect shikonin-induced apoptosis. **a** Cells were transfected with control siRNA or *RIP* siRNA. After 6 h of transfection, the cells were incubated with 3 or 6 µM shikonin for 3 h before analysis by western blotting. The cell lysates were subjected to 10 or 15% SDS-PAGE to measure the expression of the indicated proteins. Immunoblots are representative of at least two independent experiments. **b** Necroptosis was evaluated by annexin V/propidium iodide staining. **c** Values are presented as mean ± SD of three independent experiments. *p < 0.01 compared with the shikonin-treated group

To determine the impact on necroptosis, apoptosis, and autophagy, we used siRNA. The results showed that RIP1 knockdown resulted in no significant difference in the expression of cleaved PARP, cleaved caspase-3, and LC3 (Fig. 5a). However, annexin V/PI staining showed that *RIP1* siRNA-transfected cells treated with shikonin did not affect apoptosis compared to control cells transfected with scrambled siRNA (Fig. 5b, c).

### Autophagic inhibitors augment necroptosis induced by shikonin in A549 cells

**Fig. 6:**
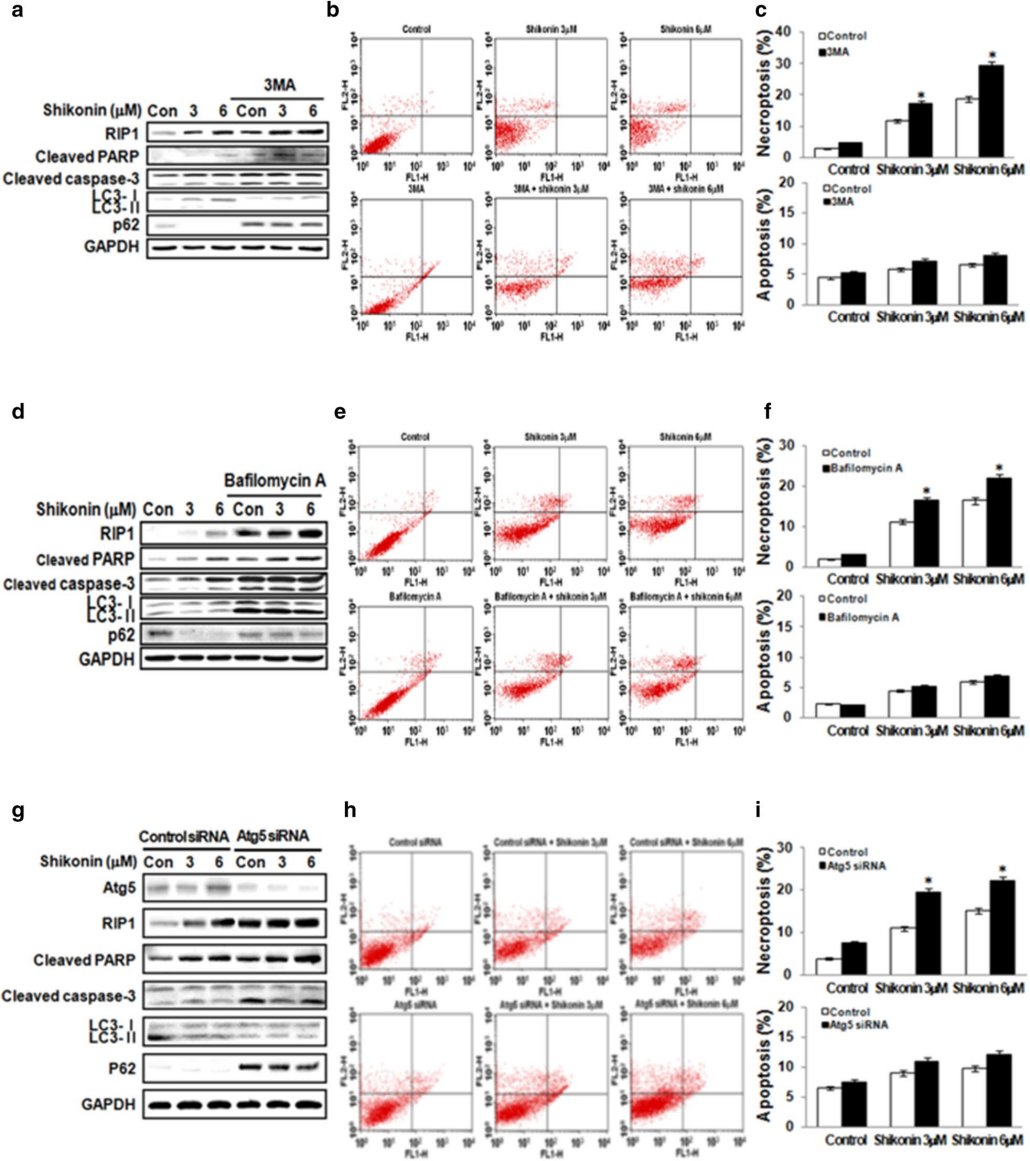
Inhibition of autophagy increases shikonin-induced necroptosis. Cells were treated with 3 or 6 µM shikonin in the presence or absence of autophagy inhibitors, i.e., 1 mM 3-MA (**a**), 50 nM bafilomycin A (**d**), and *ATG5* siRNA (**g**). The cell lysates were subjected to 10 and 15% SDS-PAGE to measure the expression of indicated proteins. **b**, **e**, **h** Necroptosis was evaluated by annexin V/propidium iodide. **c**, **f**, **i** Values are presented as mean ± SD from three independent experiments. *p < 0.01 compared with the shikonin group

To investigate the relationship between shikonin-induced necroptosis and autophagy, cells were treated with shikonin in the presence of autophagic inhibitors. The inhibitor 3-MA, which inhibits early autophagic events, decreased the levels of LC3B and increased the levels of p62 in A549 cells. In addition, 3-MA increased the expression of RIP1, cleaved PARP, and caspase-3 (Fig. 6a). Annexin V/PI staining showed that the inhibition of shikonin-induced autophagy by 3-MA significantly enhanced necroptosis in A549 cells compared to that in cells treated with shikonin alone. However, 3-MA had no effect on apoptosis in A549 cells (Fig. 6b, c). We next examined autophagic flux using bafilomycin A, a vacuolar type H^+^-ATPase inhibitor that inhibits lysosome acidification and autophagosome–lysosome fusion. As determined by an immunoblot analysis, bafilomycin A increased the levels of LC3B compared to those in control cells. We observed that the inhibition of autophagy by bafilomycin A increased shikonin-induced RIP1 and the cleaved forms of caspase-3 and PARP (Fig. 6d). Annexin V/PI staining showed that the inhibition of shikonin-induced autophagy by bafilomycin A enhances necroptosis compared to that of cells treated with shikonin alone, although there was no effect on the apoptosis of A549 cells (Fig. 6e, f).

To rule out non-selective effects of chemical inhibitors, we then examined the effect of shikonin-induced autophagy in *ATG5* siRNA-transfected cells. Silencing *ATG5* accelerated necroptosis and decreased autophagy flux, as indicated by increases in both RIP1 and the cleaved forms of caspase 3 and PARP as well as a decrease in the levels of LC3B compared to those in cells transfected with control scrambled siRNA (Fig. 6g). Annexin V staining showed that shikonin-induced autophagy significantly enhanced necroptosis in *ATG5* siRNA-transfected cells compared to control scrambled siRNA. However, again, there was no effect on the apoptosis of A549 cells (Fig. 6h, i).

## Discussion

In the present study, we attempted to address the intricate relationship between autophagy and necroptosis, focusing on the roles of autophagy in necroptosis by examining the effects of shikonin treatment. We demonstrated an anti-tumor effect of shikonin and that suppressing autophagy enhances shikonin-induced necroptosis in A549 cells. Necroptosis has been shown to be dependent on RIP3, which is activated following phosphorylation by the serine/threonine kinase RIP1 [19]. RIP1 kinase activity is crucial for necroptosis [20]; the allosteric RIP1 kinase inhibitor (necrostatin-1) inhibits death receptor-induced necroptosis in various cellular models [21]. In our study, we found that treatment with shikonin significantly increased the levels of the RIP1 protein in a concentration-dependent manner. These results, in accordance with previous data [13], indicated that shikonin induces cell death in A549 cells via the RIP1-dependent necroptosis pathway.

Necroptosis is a type of regulated cell death characterized by the loss of plasma-membrane integrity, organelle and cell swelling, and by consequent cell lysis [22]. Although the exact machinery controlling necroptosis is not completely understood, several key signaling molecules downstream of the death receptor have been identified, including RIP1 [23], RIP3 [24, 25], and JNK [26]. Several key pro-apoptosis factors have also been identified as important negative regulators of TNF-induced necroptosis. For instance, FADD and caspase 8, two essential components in the extrinsic apoptosis pathway are known to suppress necroptosis via the cleavage of RIP1 [27], while cIAP is able to block both apoptosis and necroptosis [28].

Similar to the relationship between autophagy and apoptosis, there is evidence suggesting that autophagy is capable of either promoting [29], suppressing [30, 31], or having no effect on necroptosis [32]. A combination of rapamycin and the glucocorticoid dexamethasone triggers autophagy-dependent cell death, with characteristic features of necroptosis in acute lymphoblastic leukemia cells, suggesting that autophagy promotes necroptosis in this particular system [29]. Basit et al. [33] demonstrated that recruitment of FADD to Obatoclax-induced autophagosomes directly activated RIPK1 and RIPK3, and that, importantly, caspase inhibition was not required for necroptosis in this setting. In contrast, the majority of studies have tended to demonstrate that autophagy is able to inhibit necroptosis in various systems, such as L929 cells, lymphocytes, or human cancer cells stimulated by TNFα, antigens, or starvation [30, 34]. At present, the molecular links between autophagy and necroptosis in these studies remain elusive. Here, we used zVAD, widely used as a general caspase inhibitor, as an example to further address the intricate relationship between autophagy and necroptosis. Holler et al. [21] reported that zVAD greatly sensitizes L929 cells to TNFα-induced necrosis, suggesting that zVAD itself possesses pro-necrotic function in addition to the inhibition of caspases. We also showed that zVAD induces necroptosis since zVAD-induced cell death is inhibited by necrostatin 1 via the suppression of RIP1.

In addition to necroptosis, PARP-mediated cell death is another important form of programmed necrosis. PARP is an energetically expensive process, causing the rapid deletion of intracellular ATP and eventually necrotic cell death [35]. PARP-mediated necrotic cell death also involves the translocation of apoptosis-inducing factor from mitochondria to nuclei in response to DNA damage [36]. PARP is readily activated by DNA damage, mainly DNA strand breaks, in response to oxidative stress or DNA-damaging agents [37]. Since autophagy is inducible by the disturbance of cellular energy homeostasis, the involvement of autophagy in PARP-mediated cell death has attracted substantial attention. Our results showed that autophagic inhibitors increase the expression of cleaved PARP, suggesting that the induction of autophagy by shikonin is related, at least in part, to PARP-mediated cell death in A549 cells.

Shikonin, a naturally occurring naphthoquinone, has been reported to induce necroptotic cell death in cancer cells, including those resistant to drugs and apoptosis [23, 24]. However, previous studies have not examined whether necroptosis is also induced in NCSCL cells. Flow cytometry with Annexin V and PI double staining is an effective method to quantitatively distinguish between necrosis and apoptosis. The flow cytometry data in this study showed that incubation with Nec-1 before exposure to shikonin substantially reduced the number of necrotic cells, while Z-VAD-FMK, a general inhibitor of apoptosis, had no obvious effect. Despite previous results indicating that Nec-1 suppresses apoptosis in a mouse traumatic brain injury model, it is generally considered a specific inhibitor of necroptosis and has been used to differentiate necroptosis from apoptosis. Our results showed that Nec-1 did not inhibit apoptosis caused by shikonin.

In the present study, A549 cells treated with the autophagy inhibitors 3-MA and bafilomycin A displayed increased expression of cleaved PARP, caspase-3, and RIP1, suggesting that autophagy serves a protective role. These findings are further supported by our observation that shikonin-induced necroptosis was significantly increased in ATG5-silenced A549 cells. We also observed that pretreatment with 3-MA was associated with a decrease in LC3B formation and an increase in p62 levels, essentially reversing the effect of shikonin and blocking autophagy. Accumulation of p62 facilitates autophagic clearance [38], and evidence indicates that p62 levels are regulated by autophagy and accumulate in autophagy-deficient cells [39]. We found that pretreatment with bafilomycin A, which blocks subsequent autophagosomal degradation, increased the formation of LC3B in A549 cells after treatment with shikonin. Cross-talk occurring between these signaling pathways is not entirely clear and requires further investigation.

## Conclusions

In conclusion, our in vitro and in vivo data indicate that the cytotoxicity of shikonin in A549 cells mainly occurred via the induction of necroptosis and autophagy. The inhibition of shikonin-induced autophagy enhanced necroptosis as well as PARP-mediated cell death, suggesting that shikonin is a potential therapeutic agent against NSCLC cells. It is also reasonable to speculate that the modulation of the anti-necrosis function of autophagy should be considered a novel preventive or therapeutic approach for NSCLC.

## Abbreviations

NSCLC: non-small cell lung cancer
3-MA: methyladenine
PCD: programmed cell death
TNF: tumor necrosis factor
MTT: 3-(4,5-dimethyl-2-thiazolyl)-2,5-diphenyl-2*H*-tetrazolium bromide
PI: propidium iodide
PARP: poly(ADP-ribose) polymerase
HRP: horseradish peroxidase
ECL: enhanced chemiluminescence
AVOs: acidic vesicular organelles
SDS-PAGE: sodium dodecyl sulfate-polyacrylamide gel electrophoresis
siRNA: small interfering RNA
GFP: green fluorescence protein
